# Changes in Serum Fatty Acid Levels During the First Year After Bariatric Surgery

**DOI:** 10.1007/s11695-015-1980-4

**Published:** 2015-11-26

**Authors:** Chenchen Lin, Villy Våge, Svein Are Mjøs, Olav Martin Kvalheim

**Affiliations:** 1 Department of Chemistry, University of Bergen, Bergen, Norway; 2 Fjordomics, Førde Hospital Trust, Førde, Norway; 3 Department of Surgery, Voss Hospital, Bergen Health Trust, Voss, Norway; 4 Center of Health Research, Førde Hospital Trust, Førde, Norway; 5 Faculty of Health Studies, Sogn og Fjordane University College, Førde, Norway

**Keywords:** Bariatric surgery, Serum fatty acid levels, Laparoscopic sleeve gastrectomy (LSG), Biliopancratic diversion with a duodenal switch (BPDDS), Ratio of eicosapentaenoic acid (EPA) to arachidonic acid (AA)

## Abstract

**Background:**

We have assessed the effects of laparoscopic sleeve gastrectomy (LSG) and biliopancratic diversion with a duodenal switch (BPDDS) on fatty acid (FA) levels in serum. In particular, we examine the impact of surgery on the ratio of the FAs eicosapentaenoic acid (EPA) to arachidonic acid (AA) which impacts, e.g., cardiovascular health. Our hypothesis is that LSG and BPDDS influence the FA levels but that BPDDS may have a more persistent impact since BPDSS superimposes intestinal malabsorption on gastric restriction.

**Methods:**

Serum samples after overnight fasting were collected 3 months and 1 day before surgery and 3 days, 3 months, and 12 months after surgery from 10 BPDDS patients and 23 LSG patients. The levels of 16 FAs were quantified by gas chromatography. Preoperative and postoperative concentrations of EPA and AA and the ratio of EPA to AA were compared by Wilcoxon signed-rank test corrected for multiple testing using false discovery rate.

**Results:**

The ratio of EPA/AA at each of the three postoperative sampling points was lower than at the two preoperative sampling points for BPDDS with *p* < 0.05 after correcting significance levels for multiple testing. For LSG, the ratio was lower with *p* < 0.05 at 3 days and at 3 months after surgery, but not after 12 months.

**Conclusion:**

Both LSG and BPDDS lower the ratio of EPA/AA significantly and below recommended values, but LSG patients resurge toward normal values after approximately 12 months, while BPDDS patients do not.

## Introduction

Bariatric surgery may impart sustained weight loss and remission of comorbidities [1–3]. The biliopancreatic diversion with duodenal switch (BPDDS) provides a large weight loss and a high remission rate for metabolic diseases by combining a reduction of the gastric volume (to create gastric restriction) with small bowel bypass (to create intestinal malabsorption). The procedure is not standardized, and different centers use different combinations of restriction/malabsorption [4]. Laparoscopic sleeve gastrectomy (LSG) was introduced as the first stage in a two-stage approach on super-obese or high-risk patients [5] but has later been established as a stand-alone bariatric procedure [6]. LSG reduces the gastric volume without rerouting the gastrointestinal tract.

Bariatric surgery has potential to adversely alter serum nutritional markers of health. Fatty acid (FA) levels in the blood may be reduced due to decreased dietary intake after LSG and BPDDS, or due to malabsorption of lipids after BPDDS. On the other hand, FA levels may be increased due to input from reverse cholesterol transport (RCT) of FAs from the patients’ own fat deposits during the weight loss process. The FAs stored in the fat deposit are not optimal from a nutritional point of view, and compensatory measures may be necessary such as omega-3 supplements. A particular concern is the levels of eicosapentaenoic acid (EPA) and the ratio of EPA to arachidonic acid (AA). Since the pioneering work of Dyerberg et al. [7] that suggested a link between the low incidence of cardiovascular events among Greenland Eskimos and their high level of serum EPA and high level of the ratio EPA/AA, numerous studies confirmed the associations between low values on these markers and the development of many diseases [8].

By comparing the FA levels in serum at several time points during the first year after surgery, we aim at exploring the effect of restriction (LSG) and malabsorption superimposed on restriction (BPDDS). In particular, we focus on the levels of EPA and the ratio of EPA to AA. A cohort of healthy nonobese individuals recruited from the same region as the obese is used as controls with the objective to examine if the obese have a serum profile different from the normal before and after surgery.

## Materials and Methods

### Participants

Thirty-six morbidly obese ethnic Norwegians of both genders that fulfilled the criteria for bariatric surgery (BMI >40, or, BMI >35 and the presence of obesity-related disease) were recruited for this investigation. Exclusion criteria were alcohol or drug abuse and active psychosis. Patients with type 2 diabetes were selected for BPDDS. Twelve patients were selected for BPDDS and 24 for LSG, but two of the BPDDS patients and one LSG patient were excluded because the FA analyses revealed that they used EPA supplements. Mean and standard deviation of BMI were 44.4 ± 8.0 and 44.7 ± 3.8 kg/m^2^ for the BPDDS and the LSG group, respectively. One hundred thirty-six healthy nonobese men and women from the same region in Norway with BMI 24.5 ± 2.8 kg/m^2^ were used as controls. EPA and DHA serum levels determined from the FA analyses and a validated questionnaire providing information about use of supplements over the last year [9] were available to eliminate controls using omega-3 supplements. Use of lipid-lowering agents was another exclusion criterion for the control group [10].

### Blood Sampling and Blood Analyses

Blood samples were collected 3 months before surgery (visit 1), 1 day before surgery (visit 2), 3 days after surgery (visit 3), and at 3 months (visit 4) and 12 months (visit 5) follow-up. For BPDDS, three patients did not provide serum at visit 4 and one at visit 5. For LSG patients, one, three, and five patients did not provide serum at visits 2, 4, and 5, respectively. All samples were taken in the morning after overnight fasting. Serum was obtained according to a standardized protocol [11] before stored at −80 °C.

### Measurement of FA Profiles

The serum samples were worked up and analyzed by means of a minor modifications to the procedure described in Lin et al. [11]. Total amounts of 16 FAs in each sample were quantified as micrograms per gram sample.

### Statistical Analysis

The dataset with 16 FAs, total fatty acid (TFA), and saturated fatty acid (SFA) concentrations, and the ratio of EPA/AA were subjected to statistical analysis. This dataset includes the majority of FAs that are considered biologically important except docosapentaenoic acid (DPA) and 22:5 n-6 which were removed from the analysis due to coelution with major interferents.

Means and standard deviations were calculated for the two groups of patients at the five visits. Wilcoxon nonparametric signed-rank test [12] was applied to assess the statistical significance of differences between preoperative FA levels and levels 1 year after surgery at *p* = 0.05 after correction for multiple testing by calculating the false discovery rate (FDR) [13]. Furthermore, pair-wise comparisons at all sampling points were performed for EPA, AA, and the ratio of EPA/AA. A two-tailed *t* test [14] was used to compare the omega-3 FA levels of obese with nonobese subjects.

Correlation coefficients for pairs of visits were used to study the changes for BPDDS and LSG at the five sampling points. The analysis was performed by use of Sirius Version 10.0 (Pattern Recognition Systems AS, Bergen, Norway) [15].

## Results

### Comparison of Preoperative Serum FA Levels of Obese with Nonobese

At visit 1, the patients were advised to increase physical activity and change to a less carbohydrate-rich and more protein-rich diet [6], but this change in lifestyle had no effect on the FA levels. Thus, for both surgical groups, the FAs were similar and stable at the preoperative visit 1 and 2 (Table 1). Comparison with the group of nonobese subjects reveals that the omega-3 FAs are significantly lower in the obese patients than in the normal. By using a two-tailed *t* test, the differences between the obese and nonobese (BPDDS plus LSG) at visit 2 are significant at *p* < 0.0001 for α-linolenic acid (ALA), *p* < 0.0025 for EPA, and *p* < 0.05 for docosahexaenoic acid (DHA). Furthermore, the level of the essential FA linoleic acid (LA) is lower in the obese with *p* < 0.0001. However, after correcting for multiple testing, the difference for DHA is not statistically significant.

**Table 1:** Mean concentrations of 16 fatty acids sampled at five visits for BPDDS and LSG patients and for a nonobese control group (NCG) with BMI <30 from the same region as the patients

FA	NCG	BPDDS					LSG				
	(136)	1 (10)	2 (10)	3 (10)	4 (7)	5 (9)	1 (23)	2 (22)	3 (23)	4 (20)	5 (18)
14:0	44.4	36.1	41.8	20.2	25.6	32.5	45.9	37.4	21.3	26.0	34.8
16:0	909	873	964	977	891	745	943	893	942	915	870
16:1 n-9	16.3	14.1	16.3	11.5	16.3	16.6	16.8	16.5	11.9	14.1	16.4
16:1 n-7	74.8	86.6	104	80.1	102	82.9	108	113	90.7	98.8	80.5
18:0	304	284	302	259	260	207*	322	276	236	253	275
18:1 n-9	831	787	881	908	910	777	875	857	899	877	877
18:1 n-7	58.7	54.5	63.0	68.1	73.0	64.5	62.3	67.5	73.3	72.7	60.2
18:2 n-6 LA	1249	961	959	790	738	648*	1004	920	806	923	1044
18:3 n-3 ALA	31.1	21.2	24.4	12.4	13.6	15.4	24.4	20.5	13.1	15.5	18.2
20:3 n-6	59.3	59.9	61.9	50.7	42.3	44.5	72.6	63.3	48.3	45.8	59.0
20:4 n-6 AA	267	246	268	273	293	212*	269	273	283	322	276
22:0	38.3	38.1	34.9	32.2	25.3	17.9*	39.1	33.6	30.0	34.0	39.8
20:5 n-3 EPA	69.0	47.7	48.5	34.8	32.6	26.8*	43.2	39.4	27.3	29.9	33.2
24:0	38.8	33.6	32.8	26.2	20.3	19.0*	34.2	32.1	26.8	31.1	39.3
24:1 n-9	65.5	52.5	59.1	61.3	85.6	79.4	55.5	57.5	61.5	77.2	64.4
22:6 n-3 DHA	124	97.0	107	109	105	82.1*	95.4	98.8	98.3	104	96.3
SFA^a^	1334	1265	1375	1315	1222	1021	1384	1272	1256	1259	1258
TFA^b^	4180	3692	3967	3713	3633	2942	4011	3797	3669	3838	3844
EPA/AA^c^	0.271	0.211	0.189	0.140	0.115	0.130*	0.166	0.152	0.104	0.101	0.136

Number of serum samples available at each visit is provided in parentheses. Systematic names are used for labeling the FAs. In addition, abbreviations for the omega-3 FAs are included, i.e., α-linolenic acid (ALA), eicosapentaenoic acid (EPA), and docosahexaenoic acid (DHA), and abbreviations for two essential omega-6 FAs namely linoleic acid (LA) and arachidonic acid (AA). FA levels at visits 2 and 5 were compared for both groups of patients using Wilcoxon sign-rank test. The *p* values were corrected for multiple testing by calculating false discovery rate (FDR) [13]. FAs showing significant differences at corrected level *p* = 0.05 are indicated by an asterisk (*) and by two asterisks for *p* = 0.01

^a^Saturated fatty acids (SFA)

^b^Total fatty acid (TFA)

^c^EPA to AA ratio (EPA/AA)

### Changes in Serum FA Pattern After Surgery

At visit 3, 3 days after surgery, the omega-3 FAs, ALA, and EPA show strong reduction for both surgical groups (Table 1). For ALA, the levels increase again from 3 to 12 months for both groups, but for EPA, the reduction continues after BPDDS, and at 12 months, the reduction from the preoperative level is almost 50 %. After LSG, EPA increases from 3 to 12 months after surgery but is still almost 20 % below the preoperative level. Levels of AA and DHA do not change significantly except for the BPDDS group at visit 5 where level has decreased approximately 20 %. This is similar to the reduction in the level of TFA from 3 to 12 months in the BPDDS patients, implying that the relative concentration of DHA in serum is not changed. Nervonic acid (24:1 n-9) shows an increase in serum concentration 3 months after surgery in both BPDDS and LSG patients, but in LSG patients, it is back at baseline values after 1 year. In LSG patients, Wilcoxon signed-rank test corrected for multiple testing showed no significant differences between visits 2 and 5. Thus, the FA levels appear normalized or, at least, on a track back to preoperative levels 1 year after surgery. In the BPDDS group, however, total fatty acid (TFA) as well as saturated fatty acid (SFA) and most individual FAs in serum continue to decrease. The reduction in both TFA and SFA is approximately 16 %, and a similar decrease is observed for most of the unsaturated FAs. Individual FAs that are significantly different at *p* = 0.05 and *p* = 0.01 1 year after surgery in BPDDS patients, using Wilcoxon signed-rank test corrected for multiple testing, are marked with one and two stars, respectively, in Table 1.

**Fig. 1:**
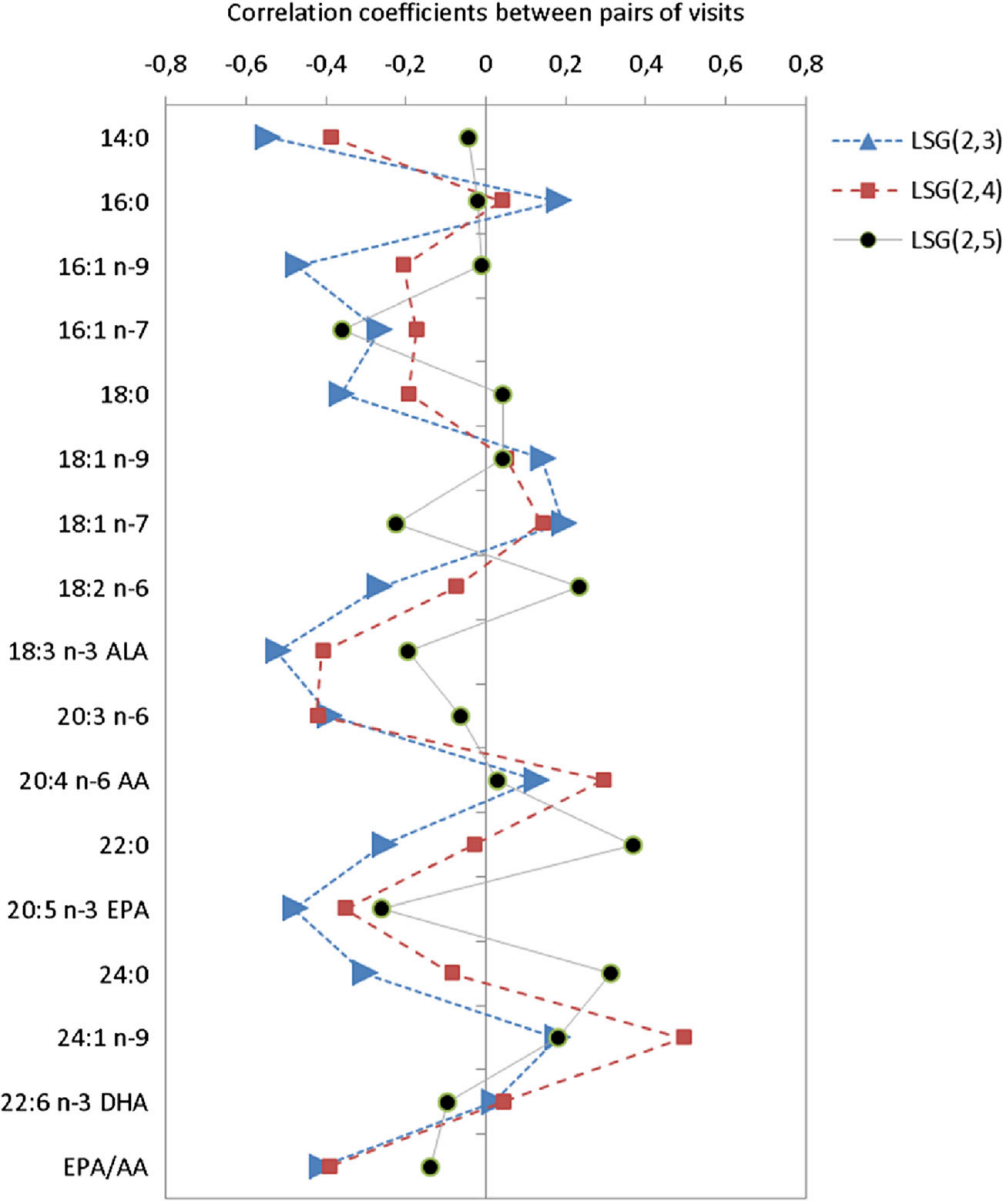
Correlation coefficients for concentrations of serum FAs between pairs of visits for LSG patients (*blue* represents visits (2,3), *red* (2,4), and *black* (2,5))

**Fig. 2:**
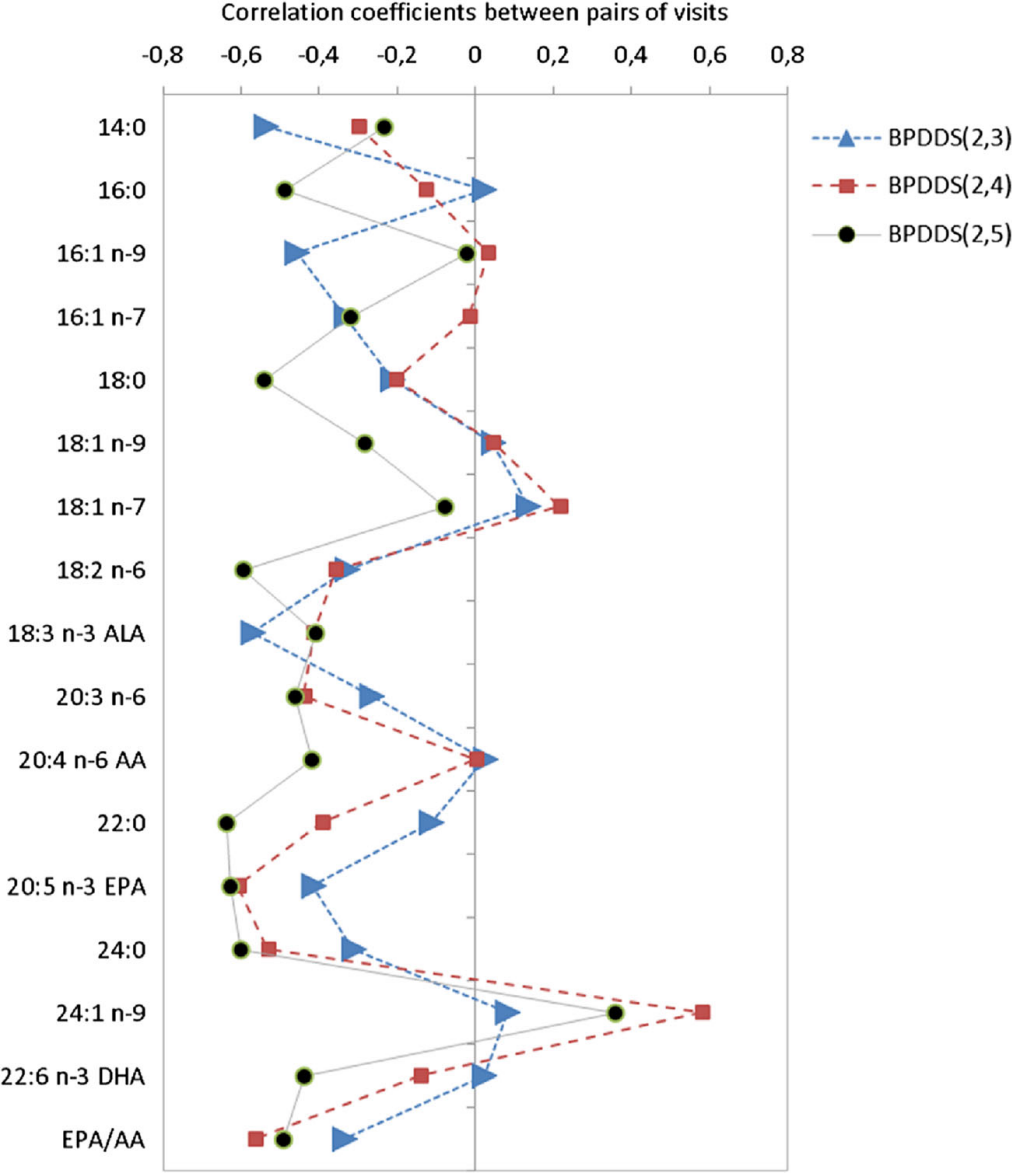
Correlation coefficients for concentrations of serum FAs between pairs of visits for BPDDS patients (*blue* represents visits (2,3), *red* (2,4), and *black* (2,5))

In order to get a more detailed picture of changes in the FA pattern after surgery, the correlation coefficients were calculated between pairs of visits. The samples at visit 2 were used as baseline for this comparison. Each patient group is considered separately to account for possible differences in the response of the metabolic system after surgery. Figure 1 shows the correlation patterns between the baseline serum concentrations at visit 2 and the three postoperative visits for the LSG patients. Three months after surgery (red curve), 14:0, ALA, 20:3 n-6, EPA, and the EPA to AA ratio display large negative correlations with visit no. 2 implying large reductions in serum levels. One year after surgery (black curve), however, most of the correlation coefficients are close to zero implying that the FA concentrations have resurged to preoperative levels. The exceptions are 16:1 n-7 with reduced levels and 22:0 and 24:0 with increased levels, but none of these changes are significant when corrected for multiple testing at *p* = 0.05, and the levels are similar to what is observed in the control group (Table 1). Figure 2 shows the corresponding plot for the BPDDS patients. Three months after surgery (red curve), the impact of surgery appears similar to the observation in the LSG group: large negative correlation coefficients for 14:0, LA, ALA, 20:3 n-6, 22:0, EPA, 24:0, and EPA to AA ratio. EPA shows the strongest negative correlation with visit no. 2 with a correlation of −0.61. However, the pattern after 1 year (black curve) is distinctly different from the LSG group with large negative correlation coefficients with visit no. 2 for 16:0, 18:0, LA, ALA, 20:3 n-6, AA, 22:0, EPA, 24:0, DHA, and the EPA to AA ratio. Thus, instead of returning to preoperative levels, changes after BPDDS are amplified after 1 year.

### Levels of Serum EPA to AA Before and After Surgery

EPA levels decreased immediately after both type of bariatric surgery (Table 2, visit 3). Over the following year, EPA levels and the ratio EPA to AA further declined for BPDDS, but after LSG, both EPA and EPA/AA increased, although levels did not reach the preoperative levels. The AA levels in the preoperative and postoperative serum samples are stable and similar for both kinds of surgeries, except for the BPDDS group at the visit one year after surgery, which shows a consistent decline for all patients. The ratio of EPA to AA follows the same trend as for EPA levels in the two patient groups since AA levels are stable. Only visit 5 for the BPDDS patients breaks this pattern by being stable from visit 4 although EPA level declines. This is due to the simultaneous drop in AA level which is relatively larger than the drop in EPA level at visit 5.

**Table 2:** Serum concentrations of eicosapentaenoic acid (EPA), arachidonic acid (AA), and the ratio of EPA to AA (EPA/AA) for visits 1–5 (mean ± standard deviation) for the two types of surgery

Visit	EPA/AA		EPA		AA	
	BPDDS	LSG	BPDDS	LSG	BPDDS	LSG
1	0.21 ± 0.10	0.17 ± 0.09	48 ± 20	43 ± 23	246 ± 99	269 ± 84
2	0.19 ± 0.08	0.15 ± 0.07	49 ± 20	39 ± 15	268 ± 80	273 ± 57
3	0.14 ± 0.06	0.10 ± 0.05	35 ± 10	27 ± 7	273 ± 117	283 ± 71
4	0.12 ± 0.04	0.11 ± 0.07	33 ± 9	30 ± 15	293 ± 88	322 ± 97
5	0.13 ± 0.04	0.14 ± 0.08	27 ± 8	33 ± 14	212 ± 51	276 ± 107

Visits 1 and 2 show preoperative levels, and visits 3–5 postoperative levels

### Statistical Assessment of Changes in Levels of EPA, AA, and EPA/AA

Since neither the levels of EPA nor AA change significantly between the two preoperative visits (Table 2), both visits were used as baseline measurements prior to surgery. Thus, we have compared the postoperative visits with both the preoperative visits to obtain a duplicate of *p* values to assess significance of changes in the postoperative phase.

For the BPDDS group, the continuous decline in the ratio EPA/AA and EPA levels after surgery is significant at *p* < 0.05 after correcting for multiple testing. The only exception is the comparison of EPA levels just after surgery with the levels 3 months before surgery (Table 3). For AA levels, there is no statistically significant change except for the levels at visit 5 when we compare with preoperative levels at hospital (visit 2), but comparison with visit 1 provides statistically insignificant, so we refrain from a definite conclusion. For the LSG patient group, changes in EPA/AA and EPA levels after surgery are similar to our findings for the BPDDS group just after surgery and 3 months later with *p* < 0.01 for EPA/AA and *p* < 0.05 for EPA whether we compare with preoperative levels at visit 1 or at visit 2. At visit 5, however, the levels are no longer statistically different from preoperative levels. This complies with the trend observed in Table 2 for the LSG group, which implies a resurgence to baseline values of EPA for this group one year after surgery. AA levels do not show statistical differences between visits for the LSG group, except for visit 4 where a statistically significant increase is observed from preoperative levels (*p* < 0.05 after correcting for multiple testing).

**Table 3:** Estimated *p* values for serum concentrations of EPA and AA and the ratio EPA/AA using Wilcoxon signed-rank test [12] to compare pairs of visits

Visit	EPA/AA		EPA		AA	
	BPDDS	LSG	BPDDS	LSG	BPDDS	LSG
(1,2)	0.401	0.596	0.589	0.308	0.250	0.749
(1,3)	0.030*	<0.001**	0.078	0.002**	0.063	0.254
(1,4)	0.020*	0.005**	0.020*	0.050	0.254	0.008*
(1,5)	0.023*	0.271	0.017*	0.174	0.091	0.889
(2,3)	0.005*	<0.001**	0.039*	<0.001**	0.589	0.208
(2,4)	0.031*	0.004**	0.032*	0.008*	0.897	0.006*
(2,5)	0.008*	0.250	0.008*	0.105	0.008*	0.190

By correcting the *p* values for multiple testing using FDR [13] and comparing the three postoperative visits with visit 1, and similarly comparing the postoperative visits with visit 2, the results for corrected significance level *p* = 0.05 and *p* = 0.01 are indicated by one and two asterisks, respectively

## Discussion

### Lower Levels of Omega-3 FAs in Obese than Nonobese

The concentration of the omega-3 FAs ALA, EPA, and DHA, and the ratio of EPA to AA are significantly lower in the morbidly obese at baseline than in the nonobese. Furthermore, the abundant essential fatty acid linoleic acid (LA) is also significantly lower in the obese than in the nonobese subjects at baseline. A possible explanation can be different dietary habits in obese and nonobese adults. However, lower concentration of serum EPA and other polyunsaturated FAs in obese subjects has been observed also in previous investigations. For instance, Micallef et al. [16] observed a significant inverse relationship between BMI and EPA and DHA in cohorts of normal, overweight, and obese adults (BMI = 33.6 ± 2.7). They proposed increased oxidative stress in obese subjects as an explanation for the reduction of polyunsaturated FAs. At baseline, the EPA to AA ratio for most of the morbidly obese patients in our study is above the recommended level of the World Health Organization (WHO) of 0.1–0.2 but below the recommended value of 0.2–0.3 in Japan [17].

### Postoperative Levels of FAs

The first year after bariatric surgery is a period of continuous weight loss. During the weight reduction process, energy consumption and body maintenance from dietary intake are supplemented by fat from the fat deposits. This fat transport affects the serum concentration of FAs since stored fat differs in composition from dietary fat. Although the composition of FAs in the fat deposits of human depends on diet and therefore may vary between populations, they are generally dominated by saturated, monounsaturated, and diunsaturated FAs with 16 and 18 carbon chains. For a Dutch general population of age 19–69, Geerling et al. [18] found the following approx. mean composition in adipose tissue for the most abundant FAs: 18:1 n-9 (42 %), 16:0 (19 %), 18:2 n-6 (15–16 %), 16:1 n-7 (5.5 %), 18:0 (3.6 %), and 18:1 n-7 (2 %). These percentages may be representative for Norwegians too, but the deposited fat of morbidly obese may be different from nonobese as observed for the baseline concentrations of EPA, ALA, DHA, and LA in serum. However, the serum levels of the other FAs of the two cohorts (Table 1) imply no significant differences at baseline. Thus, assuming that the serum levels roughly mirror the composition of the adipose tissue, obese and nonobese subjects appear similar for most of the abundant FAs at baseline. This hypothesis finds support in Table 1 which shows that the concentrations of the abundant FAs in serum are relatively stable during the weight loss process after surgery, especially for the LSG patients. For the BPDDS patients, 16:0 and 18:2 n-6 (LA) in serum gradually decline after surgery despite being abundant in adipose tissue. Of the omega-3 FAs, ALA constitutes approx. 0.7–1.1 % in adipose tissue, while AA and DHA constitute 0.3–0.5 and 0.1–0.2 %, respectively, and EPA is present in even lower amounts [18, 19]. The decline of EPA and ALA after surgery is probably the result of the reduced dietary intake accompanying the gastric restriction. The question is why this is not happening also for the polyunsaturated FAs DHA and AA. This observation is attributed to the reduction of muscle mass accompanying the weight reduction process. Both DHA and AA are abundant in muscle tissue constituting approx. 3 and 12 %, respectively, of the muscle mass [19].

### FA Profiles and Weight Loss After BPDDS and LSG

**Fig. 3:**
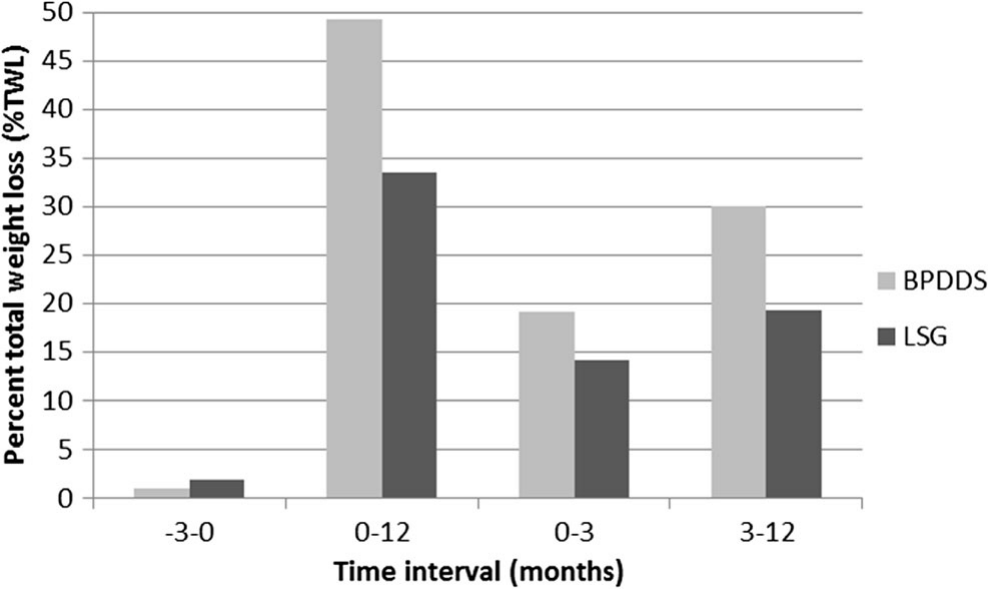
Percent total weight loss (%TWL) during preoperative (-3–0) and postoperative (0–12, 0–3, and 3–12 months) phases for BPDDS (*n* = 10) and LSG (*n* = 23) patient groups

There is a noticeable difference in the dynamics of metabolic changes between LSG and BPDDS surgery. In LSG patients, the FA levels seem to normalize approximately one year after surgery, while in BPDDS patients, the levels at 3 and 12 months imply an accelerated change. This observation may be due to malabsorption after BPDDS since this surgery also includes small bowel bypass leading to a more persistent weight reduction process (Fig. 3). One year after surgery, the % total weight loss (TWL) is 49 % in the BPDDS group and 33 % for the LSG group. Figure 3 shows that this difference increases during the last 9 months with 30 % TWL in the BPDDS group compared to 19 % in the LSG group. This may explain the differences in FA levels for the two groups after one year.

## Conclusions

Bariatric surgery has a profound effect on serum FA levels with ALA and EPA being reduced by 30–40 % from already low levels during the first year after surgery. The storage capacity of omega-3 FAs in adipose tissue is limited, so these FAs have to be supplied from continuous dietary intake. While FAs resurge toward normal levels for LSG patients, the impact appears lasting for BPDDS patients. Malabsorption may explain the persistent low levels of EPA and ALA in addition to a more enduring weight loss process. Omega-3 supplements should probably be recommended routinely after bariatric surgery until the weight has stabilized and long-term endpoints such as adverse cardiovascular events should be monitored for BPDDS patients. Longer follow-up period is needed in order to unambiguously establish if the FAs reach normal values after surgery.
